# Histo-anatomical atlas of *Garra rufa* and its thermal tolerance at human body temperature

**DOI:** 10.1038/s41598-026-67650-3

**Published:** 2026-09-14

**Authors:** Tetsuo Kon, Koto Kon-Nanjo, Shuma Nihei, Liqing Zang, Oleg Simakov, Yasuhito Shimada

**Affiliations:** 1 Department of Neurosciences and Developmental Biology, University of Vienna, Djerassiplatz 1, 1030 Vienna, Austria; 2 Department of Integrative Pharmacology, Mie University Graduate School of Medicine, 2-174 Edobashi, Tsu, Mie 5148507 Japan; 3 Graduate School of Regional Innovation Studies, Mie University, 1577 Kurimamachiya-Cho, Tsu, Mie 514-8507 Japan; 4 Mie University Zebrafish Research Center, 2-174 Edobashi, Tsu, Mie 5148507 Japan

**Keywords:** Disease model, *Garra rufa*, Histo-anatomical atlas, Teleost model, Thermal tolerance, Zebrafish, Biological techniques, Ecology, Ecology, Physiology, Zoology

## Abstract

*Garra rufa*, the doctor fish, is a small freshwater cyprinid notable for its exceptional tolerance to high temperatures, surviving at the human body temperature of 37 °C, and has emerging potential as a novel laboratory model for human cancer xenotransplantation and infectious disease research. To establish a foundation for its experimental use, we conducted comprehensive histo-anatomical analyses across major organ systems. The overall body organization and tissue architecture of *G. rufa* are broadly similar to those of zebrafish (*Danio rerio*), indicating a conserved cyprinid body plan. However, several organ systems in *G. rufa* exhibit species-specific differences from zebrafish, including a well-developed adhesive disc around the oral region, a long and coiled intestine, and distinct dark pigmentation of the peritoneum. These species-specific traits may reflect ecological and behavioral adaptations of *G. rufa*, including benthic scraping in warm, flowing habitats. Physiological assays showed that *G. rufa* maintained high survival rates and total swimming distance at 37 °C, whereas zebrafish exhibited significant mortality and a reduction in total swimming distance under the same conditions. Collectively, this study provides a comprehensive histo-anatomical atlas of *G. rufa* and documents its marked tolerance to 37 °C. Although the underlying molecular mechanisms remain unresolved, these findings establish a foundation for future mechanistic studies of high-temperature tolerance and for evaluation of its suitability as an experimental fish species.

**Supplementary Information:**

The online version contains supplementary material available at https://doi.org/10.1038/s41598-026-67650-3.

## Introduction

*Garra rufa* (Heckel, 1843), also known as the doctor fish or red garra, is a small freshwater cyprinid mainly found in rivers, hot springs, and thermal pools in the Middle East^1^. This fish has been used in ichthyotherapy, a treatment in which these fish feed on keratinized skin from human hands and feet, providing a natural exfoliation^2,3^. *G. rufa* exhibits remarkable tolerance to high temperatures, as demonstrated by its habitat in thermal waters and its application in ichthyotherapy, where the fish come into direct contact with human skin.

Small teleosts such as zebrafish and medaka are widely used as vertebrate experimental models in developmental biology, genetics, disease research, and drug discovery because of their small body size, rapid life cycle, genetic tractability, and suitability for high-throughput in vivo analyses^4–8^. Species with distinctive biological characteristics have further expanded the scope of small teleost research, including the goldfish for studies of genome duplication and phenotypic diversity^9–11^, the African turquoise killifish for aging and diapause research^12,13^, and the threespine stickleback for studies of environmental adaptation^14,15^. These examples illustrate the value of developing additional teleost models with biological properties that complement those of conventional species.

One biologically important property that differs among teleost species is thermal tolerance. Environmental temperature strongly influences biochemical reactions and physiological processes in cells and tissues^16,17^, particularly in metabolically active tissues such as the liver and visceral adipose tissue^18–20^. Other teleosts, including zebrafish, are commonly maintained at approximately 28 °C and experience stress at higher temperatures. Therefore, the high-temperature tolerance of *G. rufa* is of significant interest from the perspective of comparative physiology and evolution. Moreover, the ability to conduct experiments at temperatures near the human body temperature of 37 °C may provide a useful complementary approach for small teleost studies involving metabolic diseases, drug metabolism, human cancer xenografting, and infectious diseases. For example, in zebrafish cancer xenograft models used for drug screening, the temperature difference between the fish host and transplanted human tumor cells may affect tumor proliferation and the interpretation of experimental outcomes^21,22^.

Given its tolerance to temperatures of 37 °C or higher and the recently established chromosome-level genome assembly^23^, *G. rufa* holds great potential as a novel biomedical model complementing zebrafish^24^. In addition, the molecular mechanisms underlying the high-temperature tolerance of *G. rufa* itself may provide significant insights into the environmental adaptation strategies of vertebrates. However, detailed histo-anatomical analyses essential for developing *G. rufa* as a laboratory model, as well as direct comparisons of its survival and behavioral responses to 37 °C with those of zebrafish, have not yet been systematically addressed. We hypothesized that *G. rufa* shares a broadly conserved cyprinid histo-anatomical organization with zebrafish while exhibiting species-specific morphological features and greater physiological tolerance to 37 °C. To evaluate this possibility and establish a foundational anatomical and physiological resource for *G. rufa*, we performed comprehensive histo-anatomical analyses and compared its survival and locomotor responses at high temperature with those of zebrafish.

## Results

### Gross anatomical features

**Fig. 1:**
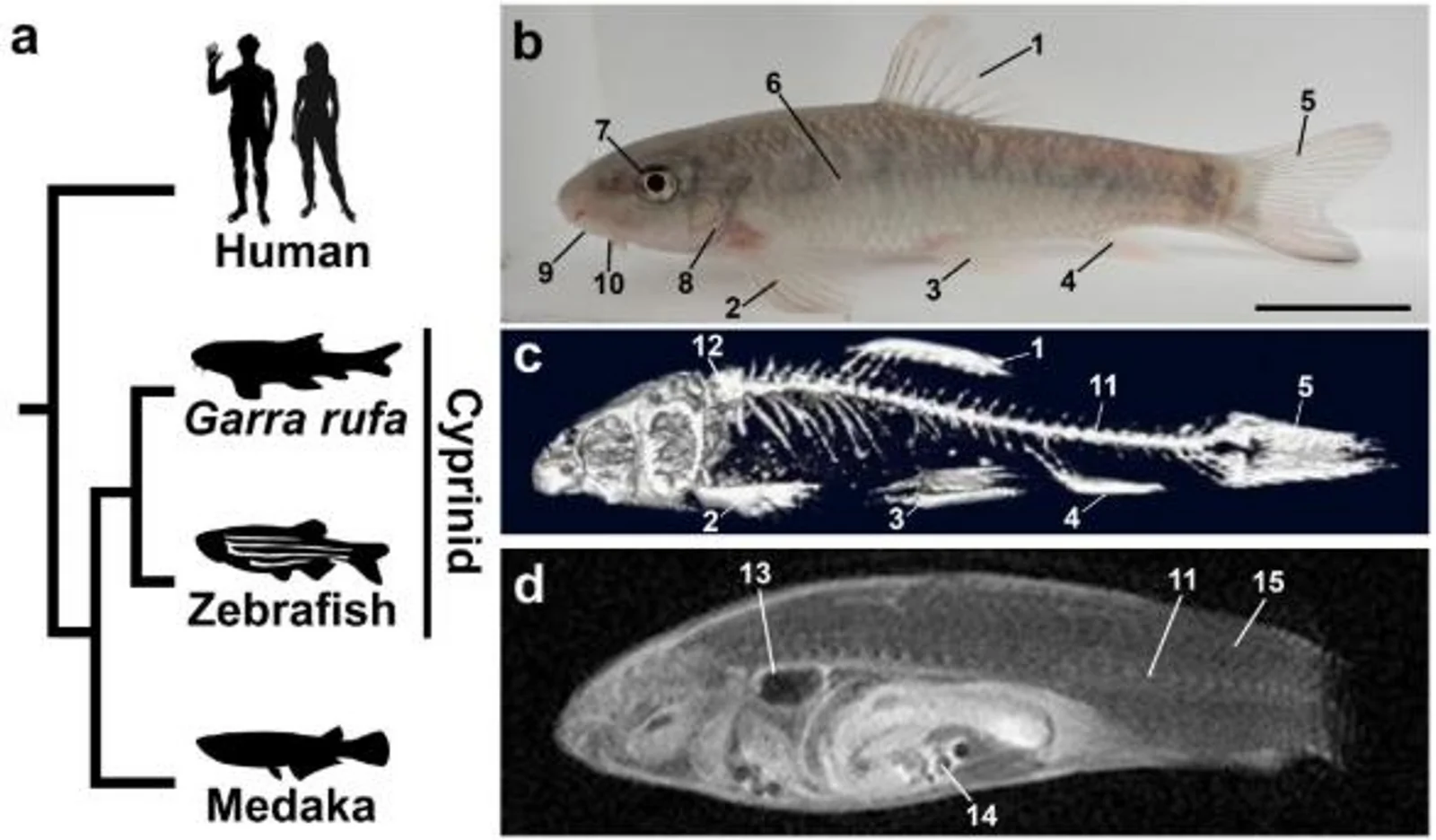
Gross anatomy of *G. rufa.* (**a**) Phylogenetic position of *G. rufa*. Animal silhouettes were obtained from PhyloPic (https://www.phylopic.org/). (**b**) External anatomy of *G. rufa*. The scale bar represents 5 mm. (**c**) Whole-body µCT image. (**d**) T1-weighted MRI scan. 1, dorsal fin; 2, pectoral fin; 3, pelvic fin; 4, anal fin; 5, caudal fin; 6, lateral line; 7, eye; 8, operculum; 9, mouth opening; 10, adhesive disc; 11, vertebral column (spine); 12, Weberian apparatus; 13, swim bladder; 14, intestinal lumen; 15, muscle tissue.

*G. rufa* belongs to the family Cyprinidae (Fig. 1a) and, like zebrafish, exhibits a streamlined body shape (Fig. 1b). The fish displays a grayish-brown body coloration, which likely enhances camouflage within its natural riverbed environment. *G. rufa* possesses dorsal, pectoral, pelvic, anal, and caudal fins that facilitate locomotion (Fig. 1b). The dorsal fin is located along the midline, while the pectoral fins are positioned laterally, posterior to the operculum (gill cover). The pelvic fins are situated ventrally near the mid-body, and the anal fin is located on the ventral side, just anterior to the caudal fin. Adult *G. rufa* at 6 months of age measured 69.5 ± 4.8 mm in total length (TL; range, 64.1–79.7 mm) and 58.7 ± 4.5 mm in standard length (SL; range, 52.1–67.6 mm), with a body weight of 4.13 ± 0.98 g (range, 3.02–6.05 g) and a Fulton's condition factor of 2.01 ± 0.16 (n = 12; Table 1). Among all morphometric traits examined, body width was the only measurement that differed significantly between the sexes, with females being wider than males both in absolute terms (13.12 ± 1.02 vs. 10.75 ± 0.93 mm) and relative to standard length (body width / SL, 0.224 ± 0.011 vs. 0.185 ± 0.013; *p* < 0.01; Table 1). Fulton's condition factor also tended to be higher in females, although this difference was not significant (2.09 ± 0.19 vs. 1.94 ± 0.08; *p* = 0.13). These observations are consistent with abdominal distension due to gravidity in females. Total length, standard length, body weight, and all fin proportions showed no significant differences between the sexes (all *p* > 0.05; Table 1). Although the difference in body width was subtle in dorsal view (Fig. S1), it was clearly resolved by quantitative measurement, providing a useful quantitative external criterion for distinguishing adult females from males under our rearing conditions. In our facility, *G. rufa* reached a standard length of approximately 5 cm by around 6 months of age, at which point the sexes could be distinguished and eggs could be obtained, indicating that sexual maturity is attained by this stage.

**Table 1:** Body size and fin proportions of adult *G. rufa* (6 months of age).

Measurement	All (n = 12)	Female (n = 6)	Male (n = 6)	*p* value
Body size				
Total length, TL (mm)	69.49 ± 4.84	69.80 ± 4.50	69.18 ± 5.58	0.82
Standard length, SL (mm)	58.69 ± 4.51	59.16 ± 4.38	58.22 ± 5.02	0.59
Body weight (g)	4.13 ± 0.98	4.39 ± 1.04	3.87 ± 0.94	0.39
Fulton's condition factor, K	2.01 ± 0.16	2.09 ± 0.19	1.94 ± 0.08	0.13
Body width (mm)	11.93 ± 1.55	13.12 ± 1.02	10.75 ± 0.93	0.008 *
Body width / SL	0.20 ± 0.02	0.22 ± 0.01	0.19 ± 0.01	0.008 *
Fin proportions (relative to SL)				
Dorsal-fin base length / SL	0.20 ± 0.03	0.20 ± 0.03	0.19 ± 0.03	0.39
Dorsal-fin height / SL	0.19 ± 0.02	0.19 ± 0.01	0.19 ± 0.02	0.82
Pectoral-fin length / SL	0.20 ± 0.01	0.19 ± 0.01	0.20 ± 0.01	0.59
Pelvic-fin length / SL	0.16 ± 0.01	0.17 ± 0.01	0.16 ± 0.01	0.18
Anal-fin base length / SL	0.09 ± 0.01	0.09 ± 0.01	0.09 ± 0.01	0.94
Anal-fin height / SL	0.13 ± 0.01	0.13 ± 0.01	0.13 ± 0.01	0.94
Caudal-fin length / SL	0.19 ± 0.02	0.19 ± 0.02	0.20 ± 0.01	0.31

Values are presented as mean ± standard deviation. Fin dimensions were normalized to the standard length (SL) of the same individual; Fulton's condition factor was calculated as K = 100 × body weight (g) / SL (cm)^3^. Differences between sexes were assessed using the Mann–Whitney U test. * *p* < 0.05. Except for body width, no measurement differed significantly between sexes. Base length, length of the fin base; height, length of the longest fin ray.

To obtain an overview of the skeletal organization of *G. rufa*, we performed whole-body micro-computed tomography (µCT) imaging and assessed gross skeletal features visible in the reconstructed images (Figs. 1c and S2). The µCT images visualized the vertebral column and the skeletal supports of the fins. The anterior vertebrae immediately behind the skull were modified and differed in form from the posterior abdominal vertebrae, consistent with their incorporation into the Weberian apparatus characteristic of otophysan fishes (Fig. S3). From these images, *G. rufa* was found to have 29–30 vertebrae (29 in two of three specimens and 30 in one), excluding the anterior vertebrae incorporated into the Weberian apparatus. Magnetic resonance imaging (MRI) visualized the spatial relationships among the internal organs, such as the intestine and swim bladder, as well as the presence of air-filled lumens (Figs. 1d and S4). A lateral line system extends along the flanks, allowing the fish to detect water currents and vibrations, thereby aiding in navigation and prey detection (Fig. 1b). The eyes are positioned laterally, providing *G. rufa* with a wide field of peripheral vision, presumably for spotting both predators and prey (Figs. 1b, S1). Notably, *G. rufa* features an inferior mouth, with the lower lip modified into an adhesive disc (Fig. 1b). This adhesive disc enables *G. rufa* to attach firmly to underwater surfaces and scrape biofilms or algae for feeding in flowing water environments^25^.

### Histo-anatomical findings

To characterize the internal anatomy of *G. rufa* in detail, we conducted gross anatomical observations of the visceral organs (Fig. S5a–l). In addition, tissue sections were prepared and stained with hematoxylin and eosin (H&E) to examine the histological architecture of each organ or tissue (Fig. S6a–e). We also utilized publicly available zebrafish virtual slides^26^ to compare conserved structures between *G. rufa* and zebrafish, and to identify morphological features unique to *G. rufa* (Fig. S7a–w and Table S1).

### Digestive tract

#### Mouth

**Fig. 2:**
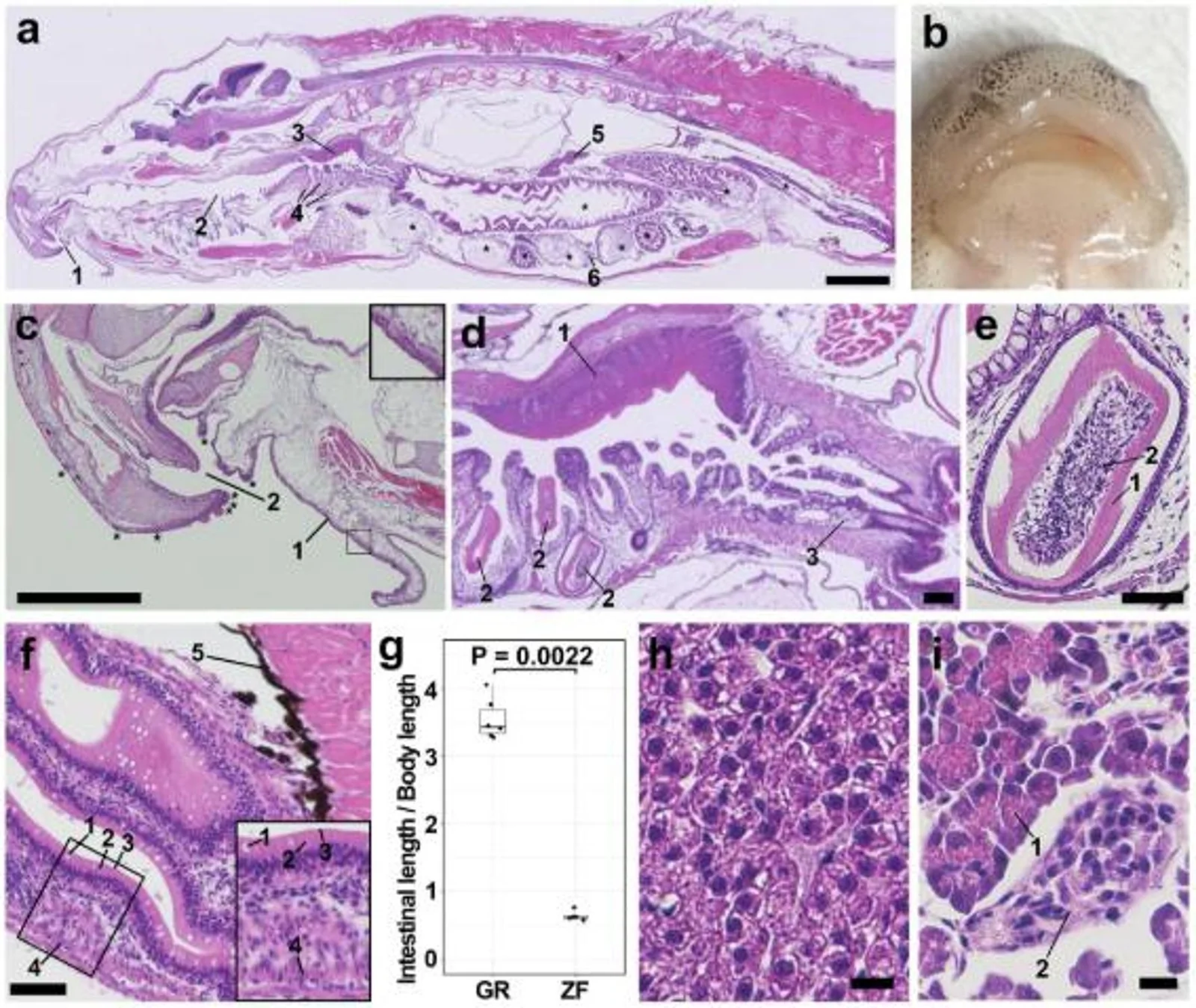
Digestive tract and glands associated with the digestive tract. (**a**) Low-magnification image showing the overall arrangement of the digestive tract. 1, mouth opening; 2, oropharynx; 3, pharyngeal pad; 4, pharyngeal tooth; 5, liver; 6, pancreas in the mesentery. Asterisks indicate the intestine. Scale bar: 1 mm. (**b**) Gross morphology of the mouth and the adhesive disc. (**c**) Sagittal section of the mouth region showing the adhesive disc. 1, adhesive disc; 2, mouth opening. The boxed area is shown at higher magnification in the inset. Asterisks indicate taste buds. Scale bar: 500 µm. (**d**) Pharynx and esophagus. 1, pharyngeal pad; 2, pharyngeal tooth; 3, goblet cells in the esophagus. Scale bar: 100 μm. (**e**) Pharyngeal tooth. 1, dentine layer; 2, pulp cavity. Scale bar: 50 μm. (**f**) Peritoneum and intestine. 1, goblet cells; 2, enterocytes; 3, brush border; 4, smooth muscle layer; 5, peritoneum. The boxed area is shown at higher magnification in the inset. Scale bar: 50 μm. (**g**) Comparison of intestinal length between *G. rufa* (GR) and zebrafish (ZF). Boxes represent the interquartile range, horizontal lines indicate the median, and whiskers extend to the most extreme values within 1.5 times the interquartile range. Individual dots represent measurements from individual fish. (**h**) Liver. Hepatocytes are densely arranged without a clear lobular or cord-like organization. Scale bar: 10 μm. (**i**) Pancreas. 1, acinar cells; 2, islet of Langerhans. Scale bar: 10 μm.

The lower lip of *G. rufa* is modified to form an adhesive disc (Fig. 2a–c). This disc is circular and features a smooth, shallow central depression, which functions as an effective structure for adhesion. This adaptation facilitates adherence to rocks and pebbles in flowing water, thereby supporting the benthic lifestyle of this fish^25^. The adhesive disc is covered by stratified squamous epithelium (Fig. 2c). In contrast, zebrafish do not exhibit such specialization of the lower lip for adhesion to surrounding substrates (Fig. S7a), which is consistent with their pelagic, free-swimming behavior rather than a benthic lifestyle^27^.

#### Oropharynx

The oropharynx constitutes the transitional segment between the oral cavity and the esophagus. Histologically, the oropharyngeal epithelium contains numerous goblet cells (Fig. 2d). A distinct pharyngeal pad is present on the dorsal wall at the posterior part of the oropharynx. The pharyngeal pad consists of a thickened mucosa with stratified squamous epithelium (Fig. 2d). Pharyngeal teeth are located ventral to the pharyngeal pad (Fig. 2d). The pharyngeal teeth exhibit a dentine layer and a central pulp cavity (Fig. 2e) and lack roots. In *G. rufa*, the pharyngeal teeth have been reported to be arranged in three rows, with a tooth formula of 2,4,5–5,4,2 (or 2,4,4–4,4,2) and hooked tips^28^. These teeth presumably work together with the pharyngeal pad to grind ingested food^29^. Zebrafish also possess pharyngeal teeth and a pharyngeal pad (Fig. S7b, c).

#### Esophagus

The esophagus extends from the oropharynx to the anterior segment of the intestine (Fig. 2d). The esophagus shows prominent mucosal folds, a feature characteristic of the highly distensible teleost esophagus, which permits the swallowing of substantial quantities of food and water^29^. In zebrafish, the esophagus similarly exhibits pronounced mucosal folds (Fig. S7b). The esophageal wall is organized into layers from the lumen outward, comprising the mucosa, the muscle layer, and the serosa (Fig. 2d). The esophageal epithelium is composed of epithelial cells and numerous goblet cells.

#### Abdominal cavity

The abdominal cavity contains organs such as the intestine, liver, pancreas, and spleen (Fig. S5a). Its surface is lined by the peritoneum. Notably, in *G. rufa*, extensive black pigmentation is observed on the peritoneal surface (Fig. S5b). Black pigmentation of the peritoneum has also been reported in other *Garra* species^30^. In contrast, the peritoneum of zebrafish is pale and lacks such extensive black pigmentation (Fig. S5l). Microscopically, the peritoneum of *G. rufa* shows extensive melanin deposition, which is consistent with the macroscopic observation (Fig. 2f).

#### Intestine

The intestine serves as the main organ responsible for digesting food and absorbing nutrients^31^. Intestinal morphology has been reported to vary greatly among teleost species depending on their dietary habits^32^. The intestine is continuous with the esophagus and is located ventral to the swim bladder (Fig. 2a). Neither *G. rufa* nor zebrafish exhibit an anatomically distinct stomach (Fig. 2a), which is consistent with the fact that cyprinids are well known to be agastric teleosts^33^. Macroscopically, the intestine of *G. rufa* is highly coiled (Fig. S5a and c). This contrasts with the macroscopically simple and largely straight intestine of zebrafish, which possesses only two major bends defining the conventional anterior, mid, and posterior segments^29,34^. When intestinal length was normalized to body length, relative intestinal length was greater in *G. rufa* than in zebrafish (two-sided Mann–Whitney U test, *p* = 0.0022; Fig. 2g). The median relative intestinal length was 3.4 in *G. rufa* (interquartile range, 3.3–3.7) and 0.62 in zebrafish (interquartile range, 0.62–0.63). Adipose deposition in the mesentery is also markedly prominent in *G. rufa* (Fig. S5c).

Histologically, whole-body sagittal and frontal sections showed multiple intestinal cross-sections within the abdominal cavity, consistent with the highly coiled organization of the intestine observed macroscopically (Figs. 2a, S5a, c, and S6a). The intestinal wall contains the mucosa and the muscle layer (Fig. 2f). The intestinal mucosa of *G. rufa* formed distinct folds, which were particularly evident in the anterior intestinal region near the junction with the esophagus (Figs. 2a, S6b, e). This distribution is broadly consistent with that reported in adult zebrafish, in which intestinal mucosal folds become progressively shorter along the rostrocaudal axis^35^. The mucosa comprises an epithelial layer and the lamina propria beneath it. In the epithelial layer of the mucosa, the columnar-shaped absorptive enterocytes are the most abundant cell type (Fig. 2f). Enterocytes possess brush borders, which likely facilitate nutrient absorption. Goblet cells are scattered between the enterocytes. The lamina propria consists of loose connective tissue. The muscle layer consists of two layers of smooth muscle fibers, an inner circular layer and an outer longitudinal layer, which together support intestinal movements.

### Glands associated with the digestive tract

#### Liver

The liver is a vital organ with broad physiological functions, encompassing metabolism, detoxification, and the synthesis of essential proteins^35^. Macroscopically, the liver forms flat, elongated, and branched lobes that overlay the intestine within the body cavity (Fig. S5c). Microscopically, *G. rufa* hepatocytes lack a distinct lobular or cord-like arrangement, and the portal areas are indistinct (Fig. 2h), in contrast to the well-organized lobular architecture characteristic of mammals^36,37^. The hepatocytes exhibit an irregular polygonal morphology with rounded nuclei. The liver parenchyma is compact and contains minimal connective tissue. The liver of the zebrafish displays a histological organization similar to that of *G. rufa* (Fig. S7e).

#### Pancreas

In both teleosts and mammals, the pancreas secretes digestive enzymes into the intestinal tract to facilitate food digestion and also functions as an endocrine organ by producing multiple hormones^36,37^. Unlike mammals, which possess a compact pancreatic organ^37,38^, the pancreatic tissue in *G. rufa* is diffuse, existing as scattered islands embedded within the mesenteric adipose tissue, a feature typical of other teleosts^39^. Histologically, the pancreas consists of both endocrine and exocrine components (Fig. 2i). The acinar structures of the exocrine component contain acinar cells with basophilic cytoplasm and eosinophilic zymogen granules. The islets of Langerhans are interspersed among the exocrine components (Fig. 2i).

### Cardiovascular system and blood

#### Heart

**Fig. 3:**
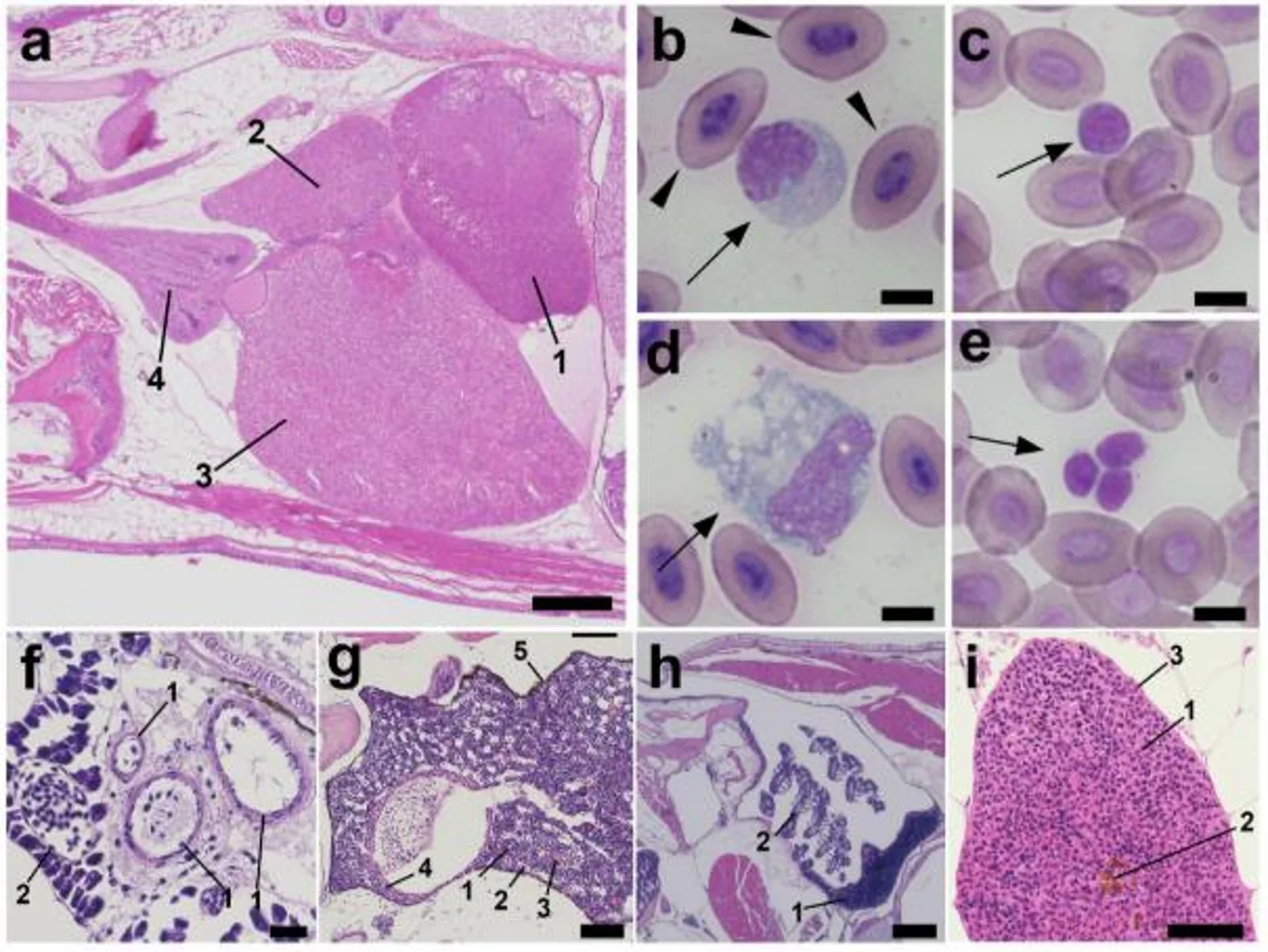
Heart, peripheral blood, and immune organs. (**a**) Heart. 1, sinus venosus; 2, atrium; 3, ventricle; 4, bulbus arteriosus. Scale bar: 200 μm. (**b**) Erythrocytes (arrowheads) and neutrophil (arrow). Scale bar: 10 µm. (**c**) Lymphocyte (arrow). Scale bar: 10 µm. (**d**) Macrophage (arrow). Scale bar: 10 µm. (**e**) Thrombocytes (arrow). Scale bar: 10 µm. (**f**) Arteriole and venule. 1, arteriole; 2, venule. Scale bar: 20 μm. (**g**) Pronephros. 1, lymphatic tissue; 2, blood sinus; 3, thyroid follicles; 4, adrenal tissue. Scale bar: 100 μm. (**h**) Thymus. 1, thymus; 2, gill. Scale bar: 200 μm. (**i**) Spleen. 1, splenic parenchyma; 2, melanomacrophage center; 3, splenic capsule. Scale bar: 50 μm.

The vertebrate heart is a continuously beating organ responsible for circulating blood throughout the body^40^. In *G. rufa*, the heart is located ventral to the esophagus and posterior to the gills (Figs. 3a and S6). Macroscopically, the heart of *G. rufa* consists of four compartments arranged in series: the sinus venosus, atrium, ventricle, and bulbus arteriosus. This structure contrasts with the four-chambered hearts of mammals^36,37,40^. The ventricular cavity contains well-developed trabeculae, and the cardiac wall is composed of myocardium formed by cardiac muscle cells (Fig. 3a). Zebrafish exhibit a cardiac structure essentially similar to that of *G. rufa* (Fig. S7g), reflecting the conserved organization of the teleost heart.

#### Peripheral blood

To evaluate the cellular composition of peripheral blood, blood smears were prepared and stained with Wright-Giemsa (Fig. 3b–e). The most abundant cell type observed was nucleated erythrocytes (Fig. 3b), a characteristic feature shared by many teleosts, including zebrafish^41^. This contrasts with the enucleated red blood cells found in mammals. In addition to erythrocytes, immune cells such as neutrophils (Fig. 3b), lymphocytes (Fig. 3c), and macrophages (Fig. 3d) were identified. Nucleated thrombocytes were also present (Fig. 3e).

#### Blood vessels

As in other vertebrates, the vascular system of *G. rufa* comprises three main types of blood vessels: arteries, veins, and capillaries. The arterial system is characterized by endothelial vessels surrounded by smooth muscle layers of variable thickness (Fig. 3f), encompassing both arteries and arterioles. The venous system consists of vessels lined by endothelium with relatively thinner smooth muscle layers, including veins and venules. Veins typically run parallel to arteries (Fig. 3f). Capillaries have the thinnest vessel walls and are distributed throughout the body, including the gills and kidneys, allowing efficient transport of oxygen and nutrients.

### Immune organs

#### Pronephros

The pronephros (head kidney) is a paired lymphohematopoietic organ located in the anterior dorsal body cavity, just posterior to the gills (Figs. 3g and S6). In teleosts, the pronephros primarily functions as a hematopoietic tissue^42^. Histologically, it contains abundant lymphocytes and granulocytes, while renal tubules and glomeruli are largely absent (Fig. 3g). The pronephros is surrounded by a thin capsule with melanin deposition (Fig. 3g). The microscopic organization of the *G. rufa* pronephros is closely similar to that of the zebrafish pronephros (Fig. S7h).

#### Thymus

The thymus consists of a pair of lymphoid organs positioned along the dorsal wall of each branchial cavity (Fig. 3h). This arrangement contrasts with that of the mammalian thymus, whose precursor cells migrate from the pharyngeal region into the thorax and fuse at the midline to form a single organ^43^. The cortex and medulla are not clearly separated in the thymus of *G. rufa*. Histologically, it consists of densely packed lymphocytes, consistent with its role as a primary lymphoid organ involved in lymphocyte development. Zebrafish possess anatomically and histologically similar paired thymus (Fig. S7i).

#### Spleen

The spleen is a compact, dark-red organ located in the abdominal cavity (Fig. S5e). Histologically, the spleen is populated by numerous immune cells, consistent with its role as a major immune organ (Fig. 3i). Melanomacrophage centers were observed within the splenic parenchyma (Fig. 3i**)**. These structures have been associated with immune responses in teleosts^44,45^. In the zebrafish reference section examined in this study, comparable melanomacrophage centers were not clearly identifiable (Fig. S7j).

### Respiratory system

#### Gills

**Fig. 4:**
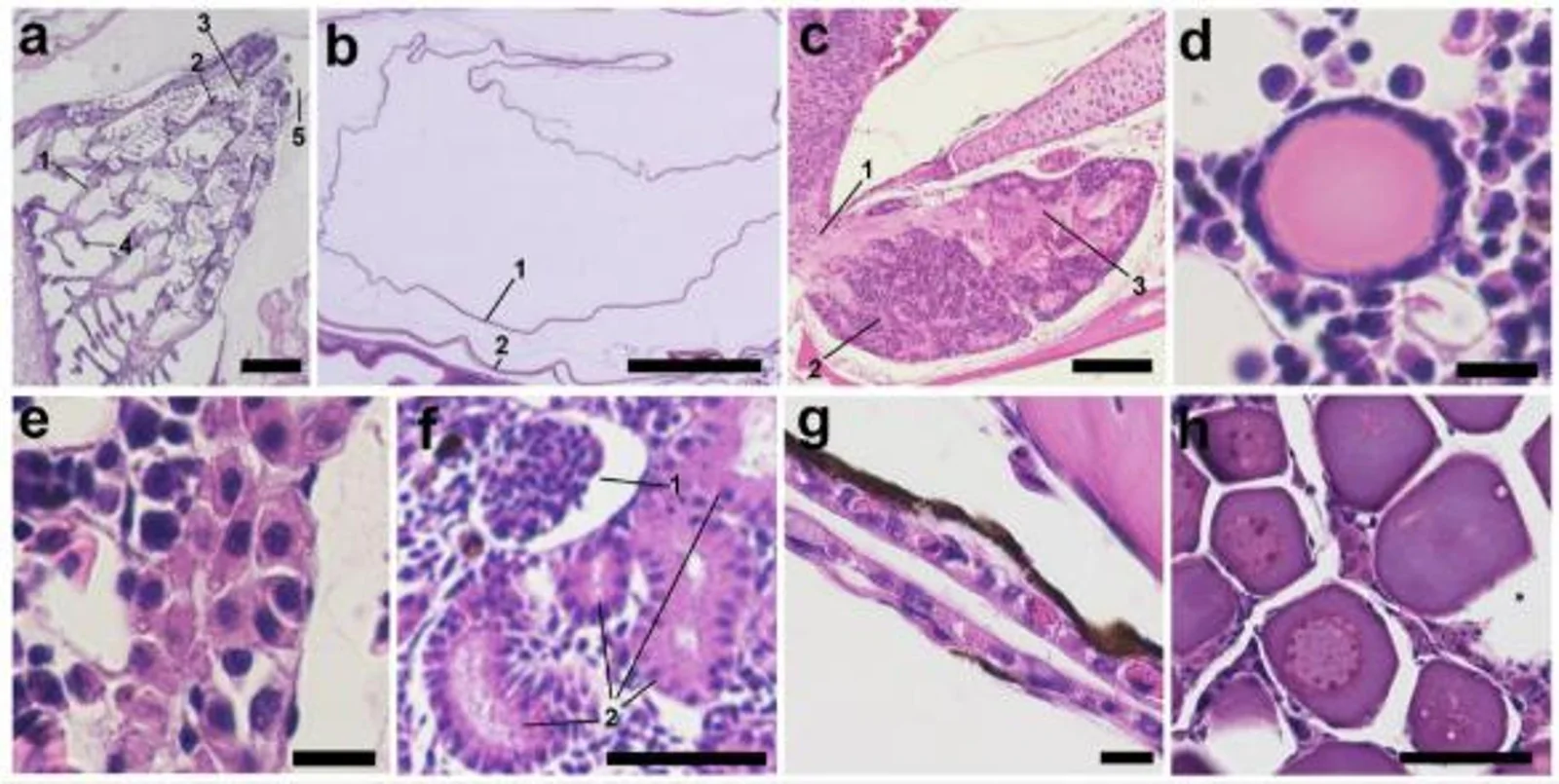
Respiratory, endocrine, urinary, and reproductive systems. (**a**) Gills. 1, gill arch; 2, primary lamella (gill filament); 3, secondary lamella; 4, gill raker; 5, gill cavity. Scale bar: 100 μm. (**b**) Swim bladder. 1, mucosal membrane; 2, outer fibrous layer. Scale bar: 500 μm. (**c**) Pituitary gland. 1, pituitary stalk; 2, anterior pituitary; 3, posterior pituitary. Scale bar: 100 μm. (**d**) Thyroid follicle. Scale bar: 10 μm. (**e**) Adrenal tissue. Scale bar: 10 μm. (**f**) Kidney. 1, glomerulus; 2, renal tubules. Scale bar: 50 μm. (**g**) Ureter. Scale bar: 10 μm. (**h**) Ovary. Scale bar: 50 μm.

The gills serve primarily as respiratory organs, but they also regulate salt and water exchange and contribute to nitrogenous waste excretion in teleosts^39,46^. The gills are symmetrically positioned in the anterior region of the oropharynx (Fig. S6). The gill of *G. rufa* is composed of gill arches, gill rakers, and primary lamellae (gill filaments) (Figs. 4a and S5i). Each primary lamella is supported by bony tissues. The gill rakers are located on the medial side of the gill arches and exhibit a serrated morphology, facilitating food filtration^29^. Microscopically, secondary lamellae arise from the primary lamellae, and the capillaries within these secondary lamellae serve as the sites of gas exchange, which is supported by unidirectional water flow across the gills (Fig. 4a).

#### Swim bladder

The swim bladder is a gas-filled organ located in the dorsal region of the body cavity (Figs. S4, S6). The swim bladder is divided into anterior and posterior chambers (Figs. S5h, S6). While the primary function of the swim bladder is buoyancy regulation, it can also function as an accessory respiratory organ that provides oxygen to the body under hypoxic conditions^47^. Multiple lines of evidence from developmental biology and gene expression studies strongly suggest that it is homologous to the mammalian lung^48^. Microscopically, the wall of the swim bladder comprises an inner mucosal membrane and an outer fibrous layer (Fig. 4b).

### Endocrine system

#### Pituitary gland

The pituitary gland is a key endocrine tissue that secretes several types of pituitary hormones^49^. The pituitary gland is located in the pituitary fossa at the cranial base (Fig. 4c). The pituitary contains two embryologically distinct components: the anterior pituitary (adenohypophysis) and the posterior pituitary (neurohypophysis). Histologically, the anterior pituitary is composed of densely packed glandular cells, whereas the posterior pituitary exhibits a neural component continuous with the pituitary stalk (Fig. 4c).

#### Islets of langerhans

The islets of Langerhans in *G. rufa* are found within the pancreatic tissue (Fig. 2i). These endocrine regions are embedded within the exocrine pancreas and are responsible for hormone production. In vertebrates, the endocrine pancreas is primarily composed of three major hormone-secreting cell types including α-cells that produce glucagon, β-cells that produce insulin, and δ-cells that produce somatostatin, together with several minor endocrine cell populations^29,35,37^. In the islets of Langerhans of both *G. rufa* and zebrafish, the endocrine cells are small to medium-sized polygonal cells with round nuclei and slightly eosinophilic cytoplasm (Figs. 2i and S7f). Subpopulations of endocrine cells are not readily distinguishable using H&E staining.

#### Thyroid

The thyroid gland of teleosts releases thyroid hormones, which stimulate many metabolic processes^39^. The thyroid of *G. rufa* is not encapsulated but consists of scattered follicles primarily located throughout the connective tissue of the pharyngeal area and within the parenchyma of the pronephros (Fig. 4d), whereas mammals exhibit clustered follicles^36,40^. In the zebrafish reference section examined in this study, thyroid follicles were not observed within the pronephric parenchyma (Fig. S7h). The thyroid follicles observed in *G. rufa* are lined with cuboidal epithelial cells and contain colloid material in the lumen.

#### Adrenal tissue

*G. rufa* does not possess a discrete adrenal gland as seen in mammals^37^. Instead, adrenal cells are scattered in association with major blood vessels and the pronephric region (Fig. 4e). These adrenal cells are presumed to consist of interrenal cells, which are considered homologous to the mammalian adrenal cortex, and chromaffin cells, which correspond to the adrenal medulla^29,35,39^. However, these two cell populations are not readily distinguishable by H&E staining.

### Urinary system

#### Kidney

In vertebrates, the kidney develops through three successive stages: the pronephros, mesonephros, and metanephros^50^. In *G. rufa*, the kidney is located in the retroperitoneal space (Figs. S5j, S6). Histologically, the kidney contains glomeruli and renal tubules (Fig. 4f). Overall, the renal histology of *G. rufa* is similar to that of zebrafish (Figs. S7h, o).

#### Ureter

The kidneys drain directly into the ureter, which forms a single tubular structure (Fig. 4g). The ureter courses along the dorsal-most aspect of the body cavity. Zebrafish exhibit a similar anatomical trajectory and histological organization of the ureter (Fig. S7p).

### Reproductive system

#### Female gonad

The ovary is a paired organ occupying much of the abdominal cavity (Fig. S6). Histologically, most visible oocytes in the ovary examined in the present study exhibited morphological features consistent with early developmental stages (stages I–II^51^) (Fig. 4h). In the zebrafish reference slide obtained from the database, the ovary contained oocytes at multiple developmental stages, a pattern consistent with asynchronous oogenesis in zebrafish^51,52^ (Fig. S7q).

### Nervous system

#### Brain

**Fig. 5:**
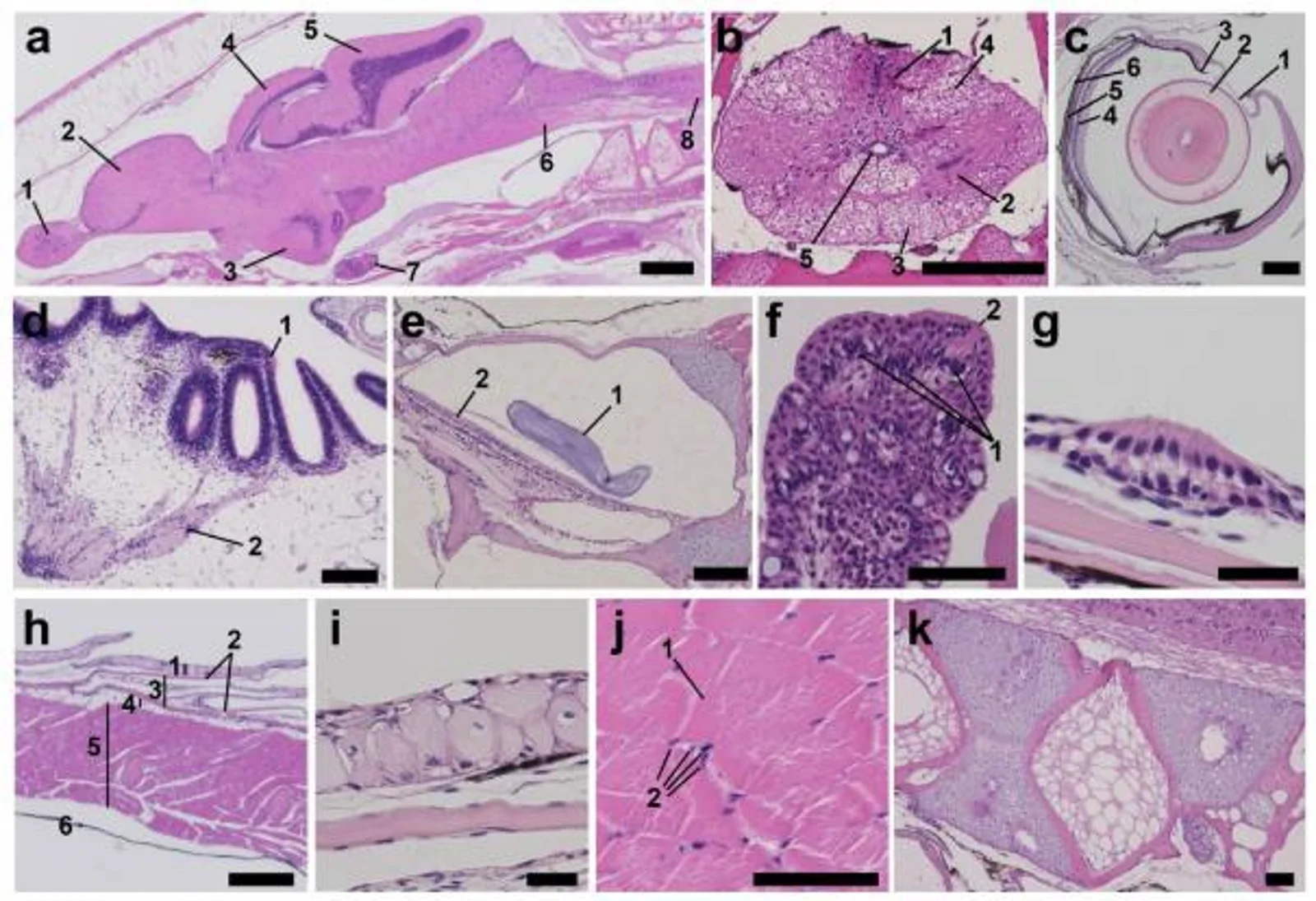
Nervous system, sensory system, skin, bone, and cartilage. (**a**) Brain and spinal cord. 1, olfactory bulb; 2, telencephalon; 3, hypothalamus; 4, optic tectum; 5, cerebellum; 6, medulla oblongata; 7, pituitary gland; 8, spinal cord. Scale bar: 500 µm. (**b**) Transverse section of the spinal cord. 1, dorsal horn; 2, ventral horn; 3, ventral funiculus; 4, lateral funiculus; 5, central canal. Scale bar: 200 µm. (**c**) Eye. 1, cornea; 2, lens; 3, iris; 4, retina; 5, retinal pigment epithelium; 6, choroid. Scale bar: 200 µm. (**d**) Olfactory sac. 1, ciliated columnar epithelial cells; 2, nerve. Scale bar: 100 µm. (**e**) Inner ear. 1, otolith; 2, sensory epithelium. Scale bar: 100 µm. (**f**) Taste buds in the pharyngeal epithelium. 1, sensory cells; 2, microvilli. Scale bar: 50 µm. (**g**) Hair cells of the lateral line. Scale bar: 20 µm. (**h**) Body wall. 1, epidermis; 2, scale; 3, dermis; 4, subcutaneous tissue; 5, skeletal muscle; 6, peritoneum. Scale bar: 200 µm. (**i**) Epidermis. Scale bar: 20 µm. (**j**) Skeletal muscle. 1, myofiber; 2, myonucleus. Scale bar: 50 µm. (**k**) Vertebra. Scale bar: 50 µm.

The brain of *G. rufa*, like that of other teleosts, is organized into major regions including the telencephalon, diencephalon, mesencephalon, metencephalon, and medulla oblongata (Figs. 5a, S5k). The telencephalon, located at the most anterior part of the central nervous system, includes the olfactory bulbs and the cerebral hemispheres. The diencephalon is located posterior to the telencephalon. The ventral region of the diencephalon is occupied by the hypothalamus (Fig. 5a). The mesencephalon is located posterior to the diencephalon and contains the optic tectum (homologous to the superior colliculus in mammals) in its dorsal region for receiving and integrating visual inputs from the optic nerve^53^. The cerebellum (part of the metencephalon) is located posterior to the optic tectum. The medulla oblongata is located at the most posterior part of the brain and is involved in vital autonomic functions such as respiration and circulation in vertebrates^54^. The overall organization of the zebrafish brain is essentially similar, comprising the same major regions (Fig. S7r).

#### Spinal cord

The spinal cord of *G. rufa* originates from the medulla oblongata and extends caudally, running within the vertebral column (Fig. 5a). Within the spinal cord, the gray matter, which is composed mainly of neuronal cell bodies, is clearly distinguishable from the surrounding white matter (Fig. 5b). The gray matter is organized into the dorsal and ventral horns. The white matter is divided into regions such as the lateral funiculus (or column) and the ventral funiculus. The central canal is located at the center of the spinal cord and is lined by ependymal cells (Fig. 5b).

### Sensory system

#### Eye

The eye of *G. rufa*, like that of other teleosts, is a laterally positioned, highly specialized sense organ^55,56^. The eyes are composed of the cornea, lens, iris, retina, retinal pigment epithelium, choroid, and sclera (Fig. 5c). Reflecting the adaptation for aquatic vision, the eye of *G. rufa* has a spherical, highly refractive lens as the primary refractive structure, similar to that of other teleosts^39^. The retina is a sheet of neural tissue lining the posterior part of the eye (Fig. 5c). The retina consists of organized neural layers and is underlain by the choroid, which supplies nutrients and absorbs excess light. Overall, the ocular structure of *G. rufa* is closely similar to that of zebrafish (Fig. S7s).

#### Olfactory sac

The primary function of the teleost olfactory sac is to detect molecules in the aquatic environment^57^. This function plays a critical role in feeding, predator avoidance, and mating behavior^58^. The olfactory sac is located on the dorsal side of the head (Fig. S6a). It consists of a pair of cavities that communicate with the outside through the external nostrils (Fig. S6e). To increase surface area, lamellae protrude into the olfactory sac (Fig. 5d). The olfactory sac is lined mainly by ciliated columnar epithelial cells (olfactory cells). In the lamina propria, olfactory nerve fibers originating from the olfactory epithelium gather into fascicles (Fig. 5d).

#### Inner ear

The inner ear functions in hearing and maintaining balance^59,60^. In the auditory system of *G. rufa*, neither an outer ear nor a middle ear is present (Fig. S6d), as in other teleosts^29^. In the otolith organs, the otoliths, which are calcified stones, lie on the surface of the sensory epithelium (Fig. 5e). The sensory epithelium consists of the ciliated hair cells. The hair cells are activated when the otolith is displaced by gravitational forces or low-frequency vibrational stimuli.

#### Taste buds

Taste buds detect chemical stimuli in the aquatic environment, facilitating food recognition and selection^61^. In *G. rufa*, taste buds are found in the oropharyngeal epithelium and on the surface of the lip (Fig. 2c), consistent with a previous histological analysis showing that external taste buds are widely distributed over the body surface and occur most frequently on the lips^62^. The sensory cells are elongated and exhibit apical microvilli projecting into the lumen (Fig. 5f). In zebrafish, taste buds are also distributed in the epithelium of the lip and the oropharyngeal epithelium, and microscopically the shape of the taste buds is similar to that of *G. rufa* (Fig. S7v).

#### Lateral line

The lateral line is a mechanosensory organ in the skin that detects water flow and vibration^63^. The lateral line canals run bilaterally along the trunk (Fig. 1b). Within the lateral line canals, there are neuromast cells, which are specialized mechanoreceptive cells (Fig. 5g). These neuromast cells are hair cells possessing sensory cilia that extend into the lumen. Water movement or vibration displaces these cilia, leading to the activation of the hair cells and transmission of signals to the central nervous system^39^.

### Musculoskeletal system and skin

#### Skin

The skin forms the outer protective barrier covering the entire body of *G. rufa* (Figs. 1b, 5h). The skin is composed of the epidermis and dermis. The epidermis of *G. rufa* is a stratified squamous epithelium. Keratinization of epidermal cells is rare except in the lip region (Fig. 2c). In the epidermis, *G. rufa* has a higher abundance of goblet cells compared to zebrafish (Figs. 5i and S7w). Scales are embedded within the dermis. While the present study documents their histological organization, a previous morphological analysis of *G. rufa* described differences in scale shape and size among body regions and between juvenile and adult specimens^64^.

#### Skeletal muscle

The bulk of the *G. rufa* body consists of segmented skeletal muscles (Figs. 1d, S4 and S6). Skeletal muscle consists of long, thin, fibrous myofibers, which are multinucleated cells (Fig. 5j). The nuclei of myofibers are located at the periphery of the fibers.

#### Bone and cartilage

Ossified tissue provides the structural framework of the body. It encases the brain for protection and forms the vertebral column along the body axis (Fig. 1c). The fins are also supported by both an endoskeleton, composed of radialia, and an exoskeleton of bony lepidotrichia, with cartilage present at the radialia (Fig. 1c). Cartilage is also observed in the gill filaments (Fig. 4a). In the intervertebral regions, the tissue represents a remnant of the notochord and a cartilaginous tissue (Fig. 5k)^65^.

#### Fins

The fin-ray counts of *G. rufa* are summarized in Table 2. Fin rays were counted at their bases: counts of the dorsal, pectoral, pelvic, and anal fins refer to branched rays, whereas the caudal-fin count refers to principal rays. The dorsal fin had 7–9 branched rays (mode 8), the pectoral fin 11–12 (mode 12), the pelvic fin 7–9 (mode 8), and the anal fin 5–6 (mode 6). The caudal fin had 19 principal rays in 11 of 12 individuals (mode 19; range 19–20), comprising 17 branched and two unbranched marginal rays, consistent with the principal-ray number typical of cyprinids^66^. In addition, the base length and height of the dorsal and anal fins and the lengths of the pectoral, pelvic, and caudal fins were measured and normalized to SL (Table 1).

**Table 2:** Fin-ray counts of adult *G. rufa* (n = 12).

Fin	Ray type	Range	Mode	Mean ± SD
Dorsal fin	Branched rays	7–9	8	7.9 ± 0.8
Pectoral fin	Branched rays	11–12	12	11.6 ± 0.5
Pelvic fin	Branched rays	7–9	8	7.9 ± 0.5
Anal fin	Branched rays	5–6	6	5.8 ± 0.4
Caudal fin	Principal rays	19–20	19	19.1 ± 0.3

To examine the skeletal organization of the caudal fin, whole-mount specimens were double-stained with alcian blue and alizarin red and imaged from the base to the distal margin (Fig. S8). The fin rays (lepidotrichia) were strongly stained with alizarin red, confirming their mineralized, bony nature, and were composed of serially arranged segments (Fig. S8b, c). Each ray was bilaterally paired (formed of two hemitrichia) and branched distally, consistent with the typical organization of cyprinid soft rays. At the base of the fin, the endoskeletal elements were strongly stained with alizarin red, consistent with their ossification in adult specimens (Fig. S8a). Clusters of large, rounded to polygonal cells in a cobblestone-like arrangement were also present in this region; these could not be reliably identified in the whole-mount preparation, since hypertrophic chondrocytes and adipocytes are difficult to distinguish under these conditions. Toward the distal margin, the rays became thinner and showed weaker alizarin red staining, indicating reduced mineralization (Fig. S8c, d). A diffuse alcian blue signal in the inter-ray regions and along the distal margin was not interpreted as cartilage, because non-specific binding to epidermal mucosubstances and residual dye could not be excluded in these whole-mount preparations.

Fin rays were counted at their bases from stereomicroscopic images of 12 adult specimens (six females and six males). Counts for the dorsal, pectoral, pelvic, and anal fins refer to branched rays; the caudal-fin count refers to principal rays, comprising 17 branched and two unbranched marginal rays. The caudal fin had 19 principal rays in 11 of 12 individuals (mode 19); a single individual had 20. Values in the “Mean ± SD” column are presented as mean ± standard deviation.

### High-temperature tolerance

**Fig. 6:**
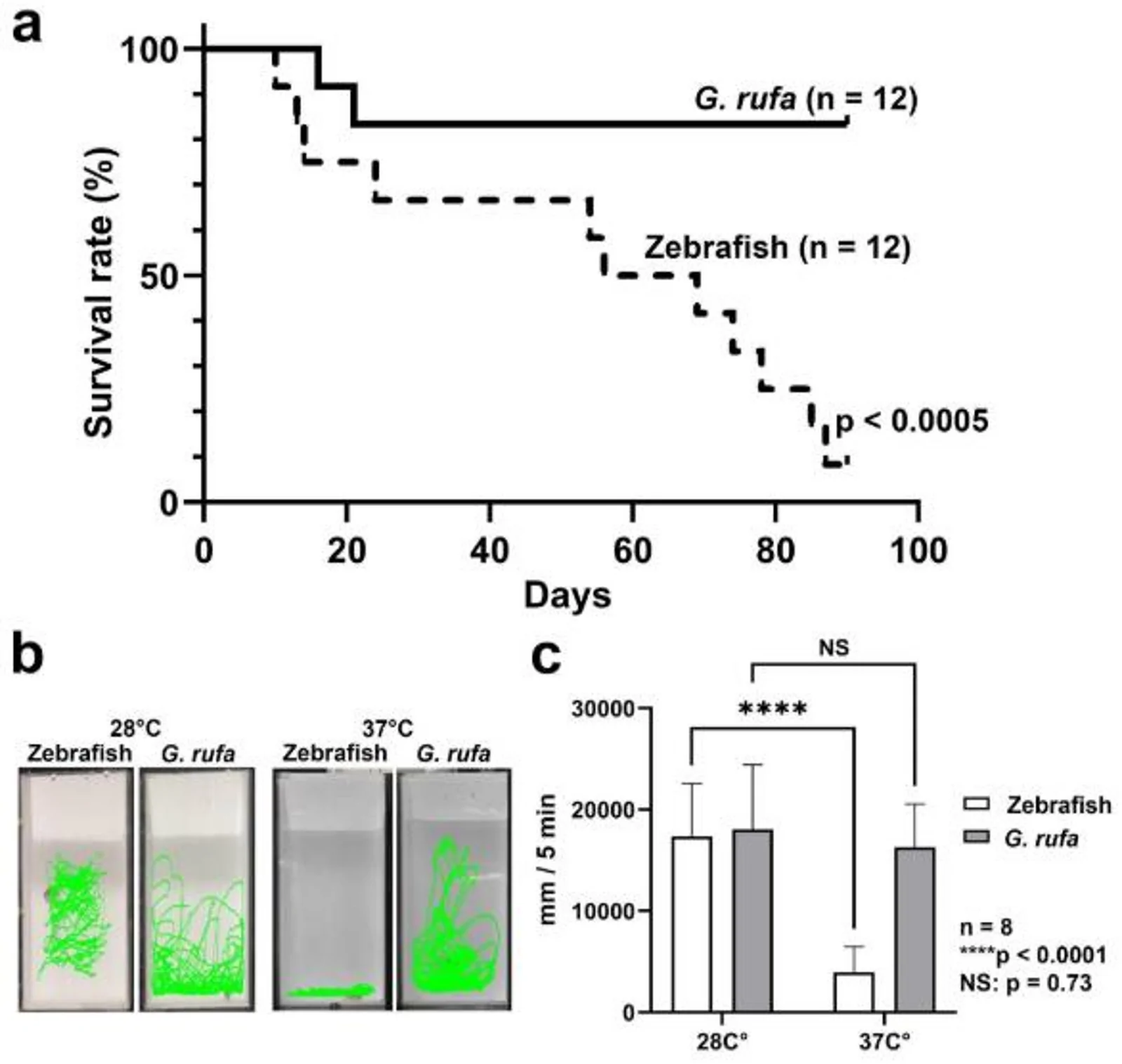
Responses of *G. rufa* and zebrafish to 37 °C. (**a**) Kaplan–Meier survival curves of *G. rufa* and zebrafish at 37 °C. While *G. rufa* maintained high survival over 90 days, zebrafish exhibited a rapid and progressive decline in survival (n = 12 for each group; log-rank test, *p* < 0.0005). (**b**) Representative swimming trajectories of zebrafish and *G. rufa* recorded for 5 min following feeding stimulation at 28 °C and 37 °C. Green lines indicate the swimming trajectories of individual fish. (**c**) Quantification of total swimming distance (mm/5 min) at 28 °C and 37 °C for each species. Data are presented as mean ± SD. n = 8; unpaired Student's *t*-test; **** *p* < 0.0001; NS, not significant.

Zebrafish are typically maintained and breed optimally at approximately 28 °C^4^, a temperature widely used as the standard rearing condition for this species, whereas *G. rufa* naturally inhabits warm rivers and thermal spring systems in the Middle East and tolerates considerably higher temperatures^24^. To quantitatively elucidate how *G. rufa* and zebrafish, a widely used model teleost, respond differently to human body temperature (37 °C), we conducted two physiological experiments. First, we evaluated the time course of survival rates of *G. rufa* and zebrafish at 37 °C. While *G. rufa* exhibited a modest decline in survival, with less than 20% mortality observed by day 20, the survival curve plateaued thereafter (Fig. 6a). In contrast, zebrafish showed a rapid decrease in survival. The difference in survival between the two species was statistically significant (n = 12, *p* < 0.0005, log-rank test).

Next, we analyzed swimming distances following feeding stimulation (Fig. 6b). In *G. rufa*, there was no significant difference in swimming distance between the 28 °C and 37 °C conditions (Fig. 6c, n= 8, unpaired Student's *t*-test, *p* = 0.73). In contrast, zebrafish exhibited a significant reduction in swimming distance at 37 °C compared to 28 °C (Fig. 6c, n = 8, unpaired Student's *t*-test, *p* < 0.0001). These results indicate that zebrafish are considerably more vulnerable to human body temperature (37 °C) in terms of both survival and total swimming distance, whereas *G. rufa* maintains comparatively high survival and swimming activity under the same conditions.

## Discussion

In this study, we performed comprehensive histo-anatomical analyses of the whole body of *G. rufa*, thereby providing an integrated description of its overall body organization. In addition, through comparison with zebrafish, a representative small teleost model organism, we characterized both morphological similarities and differences and quantitatively evaluated physiological responses to 37 °C, a temperature corresponding to human body temperature. The morphological and physiological insights obtained in this study, together with the recently reported chromosome-level genome assembly^23^, are expected to serve as foundational resources for establishing this species as a new biomedical model. Furthermore, the morphological differences revealed in this study, such as intestinal length, combined with the difference in thermal tolerance, offer valuable comparative insights into environmental adaptation and evolutionary diversification within cyprinid teleosts.

Over the past two decades, small teleosts such as zebrafish and medaka have been widely used as human disease models. These small fish models offer significant advantages for high-throughput in vivo analyses and drug screening, not only because they are subject to fewer ethical constraints than mammalian or primate models, but also because their basic anatomical and physiological features are broadly conserved across vertebrates, including humans^7,67,68^. Indeed, successful translational applications have been reported, including the discovery that prostaglandin E2 regulates hematopoietic stem cell homeostasis^69^, which led to clinical trials for cord blood transplantation^70^, and the identification of novel therapeutic candidates for Dravet syndrome using a zebrafish model^71^. Furthermore, the efficacy of existing drugs, such as ezetimibe, has been re-evaluated using a medaka model of non-alcoholic steatohepatitis^72^, with some findings advancing to clinical application.

The comprehensive histological analyses in this study, combined with the results of the preceding genome sequencing project^23^, indicate that *G. rufa* shares broad genomic and anatomical similarities with zebrafish. These similarities suggest that some research findings and experimental methodologies established in small fish models may be adaptable to *G. rufa*, although their transferability requires direct validation. Moreover, the tolerance of *G. rufa* to experimental conditions at 37 °C may offer a useful complement to existing fish models for studies requiring temperatures closer to human physiological conditions. Because many human biological processes are temperature-dependent, experiments conducted at lower temperatures may not fully reproduce responses observed at 37 °C. First, in cancer research, key processes such as the proliferation, metastasis, and angiogenesis of human tumor cells are temperature-dependent; thus, modeling these processes at lower temperatures may influence the resulting tumor phenotypes and estimates of tumor growth. Second, regarding infectious diseases, many human pathogens are adapted to proliferate at body temperature. Furthermore, the host immune response, particularly immune activation during fever, is physiologically promoted at temperatures of 37–40 °C. Consequently, ectothermic models maintained at 28 °C may not fully reproduce the dynamic interaction between the pathogen and the host. Finally, in metabolism and pharmacology, basic enzymatic reactions follow the *Q*10 temperature coefficient rule, meaning a temperature difference of approximately 9 °C (37 vs. 28 °C) can substantially affect metabolic rates. Therefore, in drug discovery, evaluating pharmacokinetics at temperatures closer to human physiological conditions may provide complementary information. However, the present study did not directly evaluate cancer xenografting, host–pathogen interactions, immune responses, drug metabolism, or pharmacokinetics. Accordingly, the applicability of *G. rufa* to these research areas requires application-specific functional validation. From these perspectives, *G. rufa* may serve as a complementary experimental fish model rather than a replacement for existing small fish models.

A limitation of the present study is that the thermal experiments were designed specifically to evaluate responses at 37 °C rather than to establish a complete temperature-response profile. Survival was evaluated at 37 °C, whereas swimming activity was compared between 28 and 37 °C; no intermediate temperature groups were included. Therefore, although our results demonstrate that *G. rufa* tolerates exposure to 37 °C better than zebrafish under the conditions tested, they do not define the temperature at which physiological impairment begins or the critical thermal maximum of either species. Previously published data nonetheless provide context for the upper thermal limit of this species. Yanar et al. reported the CTMax of *G. rufa* as 37.6, 38.1, and 39.0 °C at acclimation temperatures of 20, 24, and 28 °C, respectively, with the lowest CTMax acclimation response ratio (ARR = 0.17) among thirteen ornamental fish species examined ^73^. The chronic exposure temperature used in our study (37 °C) therefore lies just below the acute CTMax of this species (~ 39 °C), underscoring that its sustained survival and normal locomotor activity at 37 °C are physiologically notable. Importantly, CTMax reflects an acute limit determined under rapid heating, whereas our assays evaluated tolerance to prolonged exposure over days to weeks; the two metrics are complementary, and chronic thermal tolerance cannot be inferred from CTMax alone. Multiple intermediate temperatures and different exposure durations will be needed to characterize the thermal tolerance range of *G. rufa* more comprehensively, and determining the CTMax of *G. rufa* and zebrafish under our own laboratory conditions represents a further direction for future work.

Another notable feature identified in this study is the markedly elongated intestine of *G. rufa*. In contrast to the relatively simple intestinal morphology of zebrafish^74^, elongated intestinal structures have also been reported in other species of the genus *Garra*^30^, suggesting that this trait may be conserved within the genus. Previous studies have described *G. rufa* as a benthic feeder that consumes algae and biofilms in substrate-associated environments^75^. This feeding ecology provides a possible ecological context for the elongated intestine. However, the present study did not directly examine natural diet, gut contents, digestive transit time, nutrient assimilation, or the effects of diet on intestinal morphology. Therefore, the functional relationship between intestinal elongation and dietary adaptation remains hypothetical and requires direct experimental validation. While the zebrafish intestine is known to be subdivided into anterior, mid, and posterior segments, it remains unclear whether a comparable segmentation occurs during the development of *G. rufa* or whether intestinal elongation proceeds through a distinct developmental program. Investigating the intestinal development of *G. rufa* will be essential to clarify how the elongated intestine is formed and to elucidate the underlying developmental and molecular mechanisms.

Although the present study provides phenotypic evidence that *G. rufa* tolerates 37 °C better than zebrafish under the conditions tested, the underlying molecular mechanisms remain unresolved. In this context, the presence of multiple thyroid follicles in the pronephric region of *G. rufa* may be of comparative interest. However, quantitative histological and endocrine analyses will be required to determine whether this feature differs from that observed in other cyprinids or has any relationship with thermal physiology. Beyond this specific anatomical observation, a recent review highlighted the potential importance of molecular studies for understanding the high-temperature tolerance of *G. rufa*, and the chromosome-level genome assembly provides a genomic resource for such analyses^23,24^. In particular, the previous genome study identified two heat shock transcription factor (HSF) genes, 239 heat shock protein (HSP)-related genes, and 1,036 putative heat shock elements (HSEs) within the upstream regulatory regions of 944 genes^23^. Future studies should examine heat-responsive regulation in individual tissues using temperature- and time-resolved RNA-seq together with ATAC-seq to assess chromatin accessibility at HSE-containing regulatory regions. HSF ChIP-seq, or related approaches such as CUT&RUN or CUT&Tag, could further determine whether HSF proteins bind these regions in a tissue- and temperature-dependent manner, while ChIP-seq for regulatory histone modifications could characterize associated chromatin states. Integrating these molecular data with protein expression and physiological phenotypes will be necessary to identify the regulatory mechanisms underlying the high-temperature tolerance of *G. rufa*.

To further develop *G. rufa* as an experimental model organism, the establishment of laboratory-maintainable strains with uniform genetic backgrounds suitable for molecular biological analyses will be essential. In addition, the development of reliable breeding and egg collection systems, analogous to those established for zebrafish^76^, as well as the implementation of genetic manipulation techniques such as transgenesis and CRISPR/Cas9-mediated genome editing, will be critical. Moreover, extending the imaging capabilities beyond the micro-CT and MRI approaches used in this study to include tissue clearing for whole-body single-cell imaging^77,78^ and spatial transcriptomics^79^ would greatly enhance high-resolution spatial phenotyping. By establishing these experimental tools in *G. rufa* and fully utilizing the recently released chromosome-level genome sequence^23^, *G. rufa* has the potential to emerge as a complementary biomedical model that enhances the experimental utility of zebrafish.

## Methods

### Fish maintenance and sample collection

Adult *G. rufa*, originally collected from West Java, Indonesia, were bred at the National Research and Innovation Agency (North Lombok, West Nusa Tenggara, Indonesia). Zebrafish (AB line) were obtained from the Zebrafish International Resource Center (Eugene, OR, USA). Both species were maintained in laboratory aquaria at Mie University at 28 °C under a 14:10 h light–dark cycle. *G. rufa* were fed once daily with Hikari Crest Pleco (Kyorin, Himeji, Japan), while zebrafish were fed twice daily with Gemma Micro 300 (Skretting, Stavanger, Norway). All animal handling and experimental procedures were conducted in accordance with relevant institutional and national guidelines and regulations, and the reporting complied with the ARRIVE guidelines. The experimental protocols were approved by the Animal Care and Use Committee of Mie University (approval no. 2024–57). For histological and imaging analyses, healthy adult fish were anesthetized with 0.1% 2-phenoxyethanol (Fujifilm Wako Pure Chemicals, Tokyo, Japan) and euthanized by rapid cooling on ice.

### Gross anatomical observation and imaging

External morphology was observed using an SMZ745T stereomicroscope (Nikon, Tokyo, Japan) equipped with a TrueChrome II Plus Full HD HDMI camera (Biotools, Gunma, Japan). For macro imaging of large specimens, including whole-body views, a Tough TG-6 digital camera (Olympus, Tokyo, Japan) was employed in macro mode.

### Morphometric measurements

Body and fin dimensions were measured in adult specimens using a digital caliper (NGS-150KS, Spurtar; measuring range, 150 mm; resolution, 0.01 mm). For each fish, the total length (TL), standard length (SL), and the length of each fin (dorsal, pectoral, pelvic, anal, and caudal) were recorded. Measurements were performed on 12 adult *G. rufa* (six males and six females; 6 months post-fertilization).

### Fin-ray counts

Fin rays were counted from stereomicroscopic images of anesthetized (0.1% 2-phenoxyethanol) adult *G. rufa* (n = 12) acquired with the SMZ745T stereomicroscope (Nikon) and the TrueChrome II Plus camera (Biotools) described above. Rays of the dorsal, pectoral, pelvic, anal, and caudal fins were counted at their bases; the last branched ray of the dorsal and anal fins, when divided to the base, was counted as a single ray. Counts of the dorsal, pectoral, pelvic, and anal fins refer to branched rays, whereas the caudal-fin count refers to principal rays (branched rays plus the two unbranched marginal rays). Counts are reported as range, mode, and mean ± SD.

### Bone-and-cartilage double staining

The caudal fins of 6-month-old *G. rufa* were double-stained for cartilage and bone using the acid-free alcian blue/alizarin red protocol of Walker and Kimmel^80^. Specimens were fixed overnight in 4% paraformaldehyde in phosphate-buffered saline, washed, and dehydrated in 50% ethanol. Cartilage and bone were stained simultaneously by incubating the specimens overnight in acid-free double-stain solution (0.02% alcian blue 8GX, 100 mM MgCl₂, 70% ethanol, and 0.5% alizarin red S) at room temperature. Pigmentation was removed with a freshly prepared bleaching solution (1.5% H₂O₂, 1% KOH) for 20 min. Specimens were then cleared through successive changes of 20% glycerol/0.25% KOH followed by 50% glycerol/0.25% KOH, and stored in 50% glycerol/0.1% KOH at 4 °C. Stained caudal fins were observed under a BZ-X710 microscope (Keyence); whole-fin images were acquired using the image-stitching (tiling) function.

### Micro-computed tomography (µCT)

For three-dimensional skeletal visualization, whole-body µCT scanning was conducted using a Cosmo Scan CT AX system (Rigaku, Tokyo, Japan). The scanning parameters were set as follows: tube voltage, 90 kV; tube current, 88 µA; and exposure time, 4 min. The resulting images were reconstructed with an isotropic voxel size of 90 µm using Cosmo Scan CT Viewer software (Rigaku).

### Magnetic resonance imaging (MRI)

MRI was performed using a small animal MRI system (MRVivo LVA Academic Model 1506; Aspect Imaging, Shoham, Israel) equipped with a 30 mm diameter RF coil. Samples were placed on a custom-made stage consisting of a 50 mL centrifuge tube filled to 60–70% capacity with solidified 1% agarose. Images were acquired using a 2D Fast Spin Echo sequence with a flip angle of 90° and a matrix size of 128 × 256. Image reconstruction and analysis were carried out using ImageJ software (Fiji version 1.54p; NIH, Bethesda, MD, USA).

### Comparison of intestinal length between *G. rufa* and zebrafish

To quantify normalized intestinal length, we first measured the standard body length^81^, calculated as the total body length minus the caudal fin length. The entire intestine was then surgically excised, gently straightened without stretching, and measured from the anterior to posterior end to obtain the total intestinal length. The normalized intestinal length was calculated by dividing the intestinal length by the standard body length. Using this procedure, we measured the normalized intestinal length in six adult *G. rufa* and six adult zebrafish. Because the sample size was limited and the normality assumption could not be reliably established, normalized intestinal length was compared between the two species using a two-sided Mann–Whitney U test by the wilcox.test function implemented in the R version 4.1.2. P-values less than 0.05 were considered statistically significant. Box-and-whisker plots were generated using the boxplot function in R version 4.1.2. Boxes represent the interquartile range, horizontal lines indicate the median, and whiskers extend to the most extreme values within 1.5 times the interquartile range.

### Histological preparation and staining

Whole fish or dissected tissues were fixed overnight in 4% paraformaldehyde in phosphate-buffered saline (4% PFA; Falma, Tokyo, Japan) at 4 °C. The fixed samples were sent to the Division of Medical Research Support, Advanced Research Support Center (ADRES), Ehime University, where they were processed for paraffin embedding, sectioning (5 μm thickness), and routine H&E staining. Stained sections were dehydrated, mounted with coverslips, and imaged in brightfield mode using a BZ-X710 fluorescence microscope (Keyence, Osaka, Japan). When appropriate, multiple fields from the same slide or specimen were imaged to document different anatomical regions and to illustrate both low-magnification anatomical context and higher-magnification histological details; these images were used for representative histological documentation and were not treated as independent biological replicates.

### Peripheral blood smear and staining

Peripheral blood was collected from the dorsal aorta of anesthetized adult *G. rufa* using a heparinized glass capillary, following a method previously described for zebrafish^82,83^. Blood smears were prepared immediately on clean glass slides, air-dried, and stained with Wright-Giemsa Stain (ScyTek Laboratories, Logan, UT, USA) for cytological evaluation. Stained slides were examined under a BX51 light microscope (Olympus, Tokyo, Japan) equipped with an HD Lite 1080P digital camera (Tucsen Photonics, Fuzhou, China).

### Zebrafish reference histological images

Histological reference images used for comparison were obtained from the zebrafish virtual slides of the Bio-Atlas (https://bio-atlas.psu.edu/). Specific frames were cited using the “link to this” tool provided on the website. We acknowledge the Bio-Atlas project (NIH grant 5R24 RR01744), the Jake Gittlen Cancer Research Foundation, and the Pennsylvania Tobacco Settlement Fund as the sources of these materials^26^.

### Survival assay

For the evaluation of thermal tolerance, *G. rufa* and zebrafish individuals were exposed to 37 °C. The sample size was estimated based on preliminary observations indicating anticipated final survival proportions of approximately 80% in *G. rufa* and 20% in zebrafish. Assuming a two-sided alpha level of 0.05 and 80% power, approximately 10 fish per species were estimated to be required to detect this difference. We therefore used 12 fish per species to accommodate possible biological variability. The individuals were collectively maintained in a 10 L tank under controlled conditions for each group. Survival curves were generated using the Kaplan–Meier method and compared by the log-rank (Mantel–Cox) test using Prism version 10.6.1 (GraphPad Software, Boston, MA, USA).

### Swimming activity assay

After housing for 2 weeks at 28 °C or 37 °C, swimming behavior in response to feeding stimulation was evaluated. Individual *G. rufa* and zebrafish were transferred into rectangular tanks containing water equilibrated to the respective temperature (28 °C or 37 °C). Following a 10-min acclimation period, fish were fed with Gemma Micro 300 (Skretting), and swimming activity was recorded for 5 min. Videos were captured at 30 frames per second (fps) using a high-definition digital video camera (HC-V495M; Panasonic, Osaka, Japan) positioned in front of the tank. Swimming trajectories were analyzed using ToxTrac software^84^ to quantify total swimming distance (mm/5 min). Statistical comparisons between temperatures within each species were performed using an unpaired Student's *t*-test in Prism version 10.6.1 (GraphPad Software). A *p*-value less than 0.05 was regarded as statistically significant.

### Randomization and blinding

Because species identity was the predefined experimental factor, randomization with respect to species was not applicable. Formal blinding to species was not feasible because *G. rufa* and zebrafish were readily distinguishable by their external appearance. Survival was assessed using the objective binary endpoint of alive or dead. Swimming distances were quantified automatically using ToxTrac software, with identical predefined tracking parameters applied to all recordings to minimize observer-dependent measurement bias.

## Supplementary Information

Below is the link to the electronic supplementary material.

Supplementary Material 1.

Supplementary Material 2.

## Data Availability

All data generated or analysed during this study are included in this published article and its supplementary information files. The raw datasets, including micro-CT, MRI, histological images, and behavioral video recordings, are available from the corresponding author on reasonable request.
