# Supplementary material for: Histo-anatomical atlas of Garra rufa and its thermal tolerance at human body temperature
Source: Sci Rep. 2026 Sep 14;16:26799. doi: 10.1038/s41598-026-67650-3 (PMC13575154; doi:10.1038/s41598-026-67650-3)
Supplement: Supplementary file 1 [file 41598_2026_67650_MOESM2_ESM.docx]

**Supplementary Figures**

**Figure S1**

**Figure S1. Representative dorsal views of adult female (left) and male (right) *G. rufa* (6 months of age).** The female shows a wider trunk than the male, consistent with abdominal distension associated with gravidity. Body width was the only morphometric trait that differed significantly between the sexes (Mann–Whitney U test, P = 0.008; Table 1), although the difference is subtle in dorsal view.

**Figure S2**

**Figure S2. Whole-body skeletal μCT images.** Representative μCT images showing the whole-body skeletal structure from different rotational views. The red lines in the circular diagrams indicate the orientation of each view relative to the dorsal–ventral and left–right axes.

**Figure S3**

**Figure S3. Volume-rendered micro-CT images of the anterior skeleton of *G. rufa*, showing the region of the Weberian apparatus.** Two viewing angles of the same specimen (head to the left). The anterior vertebrae located immediately posterior to the skull are modified and differ in shape from the more posterior abdominal vertebrae, consistent with their incorporation into the Weberian apparatus typical of otophysan fishes (yellow arrows). Individual ossicles were not unambiguously resolved in these volume renderings.

**Figure S4**

**Figure S4. Whole-body skeletal MRI scans.**

The left column shows T1-weighted images, and the right column shows T2-weighted images.

**Figure S5**

**Figure S5. Whole-body gross anatomical findings in *G. rufa.***

(a) Intraperitoneal organs after abdominal wall resection of *G. rufa*. (b) Peritoneal cavity and its lining peritoneum of *G. rufa*. (c) Liver and intestine of *G. rufa*. Adipose deposition is observed in the mesentery. (d) Intestine of *G. rufa*. (e) Spleen of *G. rufa*. (f) Gallbladder of *G. rufa*. (g) Heart of *G. rufa*. (h) Swim bladder of *G. rufa*. (i) Gills of *G. rufa*. (j) Kidney of *G. rufa*. (k) Brain of *G. rufa*. (l) Abdominal cavity of zebrafish. Scale bars represent 1 mm.

**Figure S6**

**Figure S6. Representative whole-body histological sections of *G. rufa* in sagittal and frontal planes.** Panels a and b show sagittal sections, whereas panels c–e show frontal sections. Abbreviations: AD, adhesive disc; AF, anal fin; Bra, brain; DF, dorsal fin; Gil, gills; Her, heart; IE, inner ear; Int, intestine; Liv, liver; Mes, mesonephros; OS, olfactory sac; Ova, ovary; Pan, pancreas; Per, peritoneum; PP, pharyngeal pad; Pro, pronephros; PT, pharyngeal tooth; SB, swim bladder; SC, spinal cord; Ski, skin; Spl, spleen; Thy, thymus; Ure, ureter; VC, vertebral column. Scale bars represent 1 mm.

**Figure S7**

**Figure S7. Reference histological sections of zebrafish retrieved from the Bio-Atlas.**

Zebrafish reference histological images were retrieved from the zebrafish virtual slides of the Bio-Atlas (https://bio-atlas.psu.edu/). For details regarding the data source and attribution, refer to the Materials and Methods section. (a) Mouth. (b) Pharynx and esophagus. (c) Pharyngeal tooth. (d) Intestine. (e) Liver. (f) Pancreas. (g) Heart. (h) Pronephros. (i) Thymus. (j) Spleen. (k) Swim bladder. (l) Gills. (m) Thyroid. (n) Adrenal tissue. (o) Mesonephros. (p) Ureter. (q) Ovary. (r) Brain and spinal cord. (s) Eye. (t) Olfactory sac. (u) Inner ear. (v) Taste buds in the pharyngeal epithelium. (w) Skin. The boxed area is shown at higher magnification in the inset. Scale bars represent 100 μm.

**Figure S8**

**Figure S8. Skeletal organization of the caudal fin of *G. rufa* revealed by whole-mount alcian blue (cartilage) and alizarin red (bone) double staining.** Left, whole caudal fin in lateral view; boxed regions (a–d) are shown at higher magnification on the right. (a) Fin base, showing alizarin red–positive endoskeletal elements. Clusters of large, rounded to polygonal cells in a cobblestone-like arrangement are also present; these could not be unambiguously identified as chondrocytes or adipocytes. (b) Mid-region of the fin rays, showing alizarin red–positive, serially segmented lepidotrichia. (c) Region of ray branching. (d) Distal margin, where the rays are thinner and only weakly mineralized and alcian blue–positive tissue is more extensive.
